# Evaluation of the Bond Strength of Self-Etching Adhesive Systems Containing HEMA and 10-MDP Monomers: Bond Strength of Adhesives Containing HEMA and 10-MDP

**DOI:** 10.1155/2022/5756649

**Published:** 2022-06-10

**Authors:** Roberta Pimentel de Oliveira, Brennda Lucy de Paula, Mara Eliane Ribeiro, Eliane Alves, Hilton Túlio Costi, Cecy Silva

**Affiliations:** ^1^School of Dentistry, Federal University of Pará, Belém 66075-110, Brazil; ^2^Institutional Scanning Electron Microscopy Laboratory, Emílio Goeldi Paraense Museum, Belém 66077-530, Brazil

## Abstract

The aim of this *in vitro* study was to assess the bond strength of self-etching adhesives containing HEMA and 10-MDP monomers. Twenty-four bovine teeth were divided into three groups. Two cylinders of composite resin were made in each tooth (*n* = 16): G1-Prime and Bond Universal (control); G2-OptiBond All-in-One (HEMA); and G3-Clearfil SE (10-MDP and HEMA). After 24-hour storage in distilled water, the specimens were fixed to a universal testing machine (Kratos Equipamentos Ltda.) for the microshear test at a speed of 0.5 mm/min. A qualitative analysis of the fracture pattern was also performed using scanning electron microscopy (500× magnification). The normality of sample data distribution was determined using the Shapiro–Wilk test. The results were assessed using the Kruskal–Wallis test, and *α* level of 5% was used for the analysis. The results indicated a statistical difference (*p* > 0.05) between G3 (15.6080 MPa) and G2 (11.2180 MPa). No statistical difference was observed when G1 (14,6325 MPa) was compared with the other two groups. It was also observed that a mixed fracture pattern was predominant in all groups. The self-etching adhesive containing HEMA and 10-MDP monomers showed to be promising in increasing the bond strength between the dental substrate and the composite resin, whereas the adhesive containing only HEMA exhibited lower bond strength to dentin.

## 1. Introduction

Functional monomers are considered important components of dentin adhesives, as they regulate the interaction of the adhesive interface between dentin and dental enamel. Typically, commercial self-etching adhesives contain two specific functional monomers in their composition with the aim of increasing bond strengths, improving diffusion and penetration of other monomers, or even providing antimicrobial action. However, due to the diversity of monomer types, the literature shows a wide variation in the effectiveness of adhesives [1].

Two-hydroxyethyl methacrylate (HEMA) seems to be the most used monomer in dentin adhesive primers. Due to its low molecular weight, it was introduced with the aim of improving wettability and diffusion in the dentin structure. It is highly hydrophilic and promotes the adhesion and penetration of other monomers [2, 3]. Despite the important role of HEMA due to its hydrophilicity, wettability, and miscibility, a recent systematic review and meta-analysis compared HEMA-containing adhesive with HEMA-free adhesive systems and the authors found a similar clinical behavior [4]. Over time, high hydrophilicity promotes increased water acceptance that results in the hydrolytic degradation of the adhesive interface [5, 6]. Therefore, the addition of other monomers that help increase bond strengths and the longevity of the adhesive interface has become important.

Ten-methacryloyloxydecyl dihydrogen phosphate (10-MDP) has the ability to associate chemical adhesion with micromechanical adhesion, through binding enamel and dentin with hydroxyapatite, forming MDP-Ca salts. These salts have great stability, resistance to hydrolysis, and high longevity, providing resistance to the binding interface and stability in an aqueous medium. Thus, it was incorporated into the adhesive resin as a binding agent and as a diffusion stimulator of the adhesive. In addition, as it is an acidic functional monomer, it was also incorporated into the primer as a conditioning agent [7–9].

Hema and 10-mdp are functional monomers important for the adhesive process. However, the adhesive systems are composed of a mixture of different monomers (containing or not HEMA and 10-MDP), solvents, initiators, and nanoparticles used to promote the entire adhesive process; therefore, it is important to investigate its mechanical performance. This way, the goal of this study was to assess the bond strength of self-etching adhesive systems containing monomers HEMA and/or 10-MDP. The null hypothesis to be tested was that the presence of monomers HEMA and/or 10-MDP in the formulation of self-etching adhesives would not influence bond strength.

## 2. Materials and Methods

### 2.1. Sample Characterization

After approval by the Animal Research Ethics Committee (Protocol 4032240320; ID 001442), 24 bovine incisor teeth (*Bos taurus indicus*) were used in this study. The sample size per group (*n* = 16) was determined after a pilot test and sample calculation. The minimum difference desired in the control group was 20%, statistical power of 80% and alpha level of 5%.

After extractions, the teeth underwent a process of disinfection in 0.1% thymol solution for one week. Then, the vestibular enamel of the middle portion of the crown was assessed with a stereoscopic magnifying glass (80×), and elements that had cracks or fractures were discarded. Subsequently, the teeth selected for the study were stored in distilled water at 4°C until the moment of the preparation of the specimens and performance of the tests (ISO TS 11405: 2003).

### 2.2. Inclusion of Bovine Teeth in PVC Tubes

After cleaning and disinfecting the teeth, a section was performed at the level of the cementitious-enamel junction using a double-faced diamond disc (KG Sorensen, Cotia, SP, Brazil) under refrigeration, discarding the root portions. Then, the dental crowns were included in PVC tubes (20 mm in diameter and 1.3 cm in height) with self-curing acrylic resin (Auto Clear incolor, DentBras, Pirassununga, SP, Brazil), so that only the vestibular faces were exposed.

After the complete polymerization of the acrylic resin, the vestibular face of each specimen was grounded using a horizontal polishing machine (Aropol-E-Arotec, Cotia, SP, Brazil) with 180-grit sandpaper (3M, Sumaré, SP, Brazil) until the exposure of dentin. The dentin windows were exposed with a diameter of 1 mm [10], considering the depth of surface dentin standardized with the aid of a digital caliper (DIN 862; Mitutoyo, São Paulo, Brazil). The surfaces were then exposed to #400-and #600-grit silicon carbide sandpaper for 30 seconds each. After smoothing, the samples were washed in an ultrasonic vat (TD30 Plus; Bio-Art, São Paulo, Brazil) with distilled water for 20 minutes.

### 2.3. Experimental Groups and Preparation of Composite Resin Cylinders

With the teeth already enclosed in PVC tubes and the surface of vestibular dentin polished, the delineation of the adhesive area was performed by fixating a double-sided acid-resistant tape (Tectape, Manaus, AM, Brazil), perforated in a circular fashion (0.8 mm in diameter) with the aid of a rubber sheet perforator. Each tape received two perforations, so that each tooth had two cylinders of composite resin. Thus, each group would have eight teeth with two specimens (cylinders), totaling *n* = 16 per group. Each tooth belongs to a different group, so there was no risk of contamination from different adhesives on the same tooth. The randomization of the teeth was performed as follows: each tooth received a number from 1 to 24 which was written on the PVC tube and then the randomization sequence was generated using the Random Allocation 2.0 software (simple randomization) to allocate 8 teeth in each of the 3 groups (total 24 teeth).

The dentin bonding strategy delimited by the acid-resistant tape was performed according to the experimental group (Table 1), and the light-curing device used was LED type (D-2000; DMC, Joinvile, SC, Brazil) at 1100 mW cm^2^. Prior to the light curing of each experimental group, the light intensity of the light-curing device was measured using a digital radiometer (SDI; Victoria, Australia).

Then, the first layer of acid-resistant tape was removed. Tygon® tubes with an internal diameter of 0.8 mm and height of 0.5 mm were positioned to coincide with the circular areas demarcated by the adhesive tape. The tubes were filled with Filtek Z350 XT COR A3 composite resin (3M ESPE, Sumaré, SP, Brazil) using a 1 Ward condenser, and light curing was performed for 40 seconds. Two cylinders of composite resin were made in each tooth.

After 24-hour storage in distilled water (37°C), the tubes were removed along with the second layer of the tape using a No. 12 scalpel blade. The resin cylinders were examined with a stereoscopic magnifying glass (40×) and, if there were specimens with interface gaps or bubbles, they would be excluded and replaced for the microshear test.

### 2.4. Microshear Test

The specimens were fixed to a universal testing machine (Kratos Equipamentos Ltda., Cotia, SP, Brazil). A metal wire measuring 0.2 mm in diameter was fitted to the load cell for loading the composite resin cylinder at a speed of 0.5 mm/min. And the bond strength values were measured in megapascals (MPa).

### 2.5. Analysis Using Scanning Electron Microscopy

The fractured specimens were mounted in sample holders (stubs) and metalized for visualization under a scanning electron microscope (LEO-1430; Carl Zeiss, Oberkochen, Germany). Electron micrographs were obtained with a magnification of 500×; covering the entire fractured adhesive areas, within which failures of adhesive, mixed, composite resin cohesion, and dentin cohesion types would be estimated.

### 2.6. Statistical Analysis

The results obtained by the microshear test showed an abnormal distribution (Shapiro–Wilk test) and were analyzed with the Kruskal–Wallis test, with the results represented as median and interquartile deviation. The *α* level of significance (*p* > 0.05) was determined for all analyzes performed.

## 3. Results

**Figure 1:**
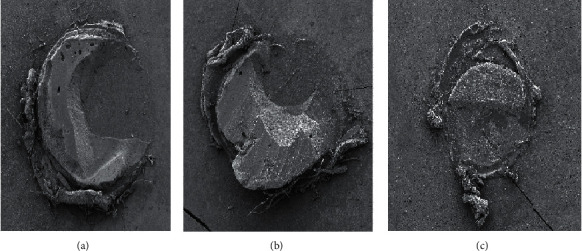
Fracture pattern of the experimental groups observed by SEM (50× magnification). (a) Mixed fracture in G1; (b) mixed fracture in G2; (c) mixed fracture in G3.

The greatest median was observed in G3 (15.6080 MPa) and the lowest in G2 (11.2180 MPa), which represented a statistical difference between these two groups. No statistical difference was observed when G1 (14.6325 MPa) was compared with the other two groups (Table 2). The prevalent fracture pattern in all experimental groups was the mixed type (Figure 1).

## 4. Discussion

The components of adhesive systems can significantly influence their interaction with the tooth structure and, consequently, have effects on adhesion. The chemical and morphological characteristics of the tooth-adhesive interface and the quality of the hybrid layer are closely related to the interaction between functional monomers and dental substrate [11, 12]. Functional monomers are considered adhesion promoters. The hydrophilic property of these monomers, such as HEMA, contributes to increasing the bond strength of adhesives to dentin, and some functional monomers can chemically bond to calcium, such as 10-MDP [13, 14].

The assessment of the physical-mechanical behavior of the interfaces established by adhesive systems and the dental substrate is important to establish a restorative prognosis. When mechanical laboratory tests are used for this purpose, many studies use the microshear test as a methodological tool to measure bond strength, both to dentin and enamel [15].

The three adhesives tested in the present study are self-etching, which may contribute to better adhesion to dentin, due to a decrease in the possibility of collagen fiber collapse, and a reduction in the risk of disparity between the etching depth and the infiltration depth of the monomers [13, 16]. Furthermore, they all have mild pH. An *in vitro* study indicated that increasing the acidity of self-etching adhesive systems did not increase the bond strength values [17]. Thus, good adhesive behavior was expected in all groups. Despite these common factors, the different functional monomers present in the formulation of these systems play an important role in the adhesive process and may influence the success and longevity of restorations. In the present study, the null hypothesis tested was rejected, as the presence of monomers HEMA and 10-MDP in the tested self-etching adhesives influenced the bond strength.

The formulation used in G1 contained other functional monomers that played an important role in the adhesion process. The functional monomers PENTA and methacrylate dihydrogen phosphate are acidic, with mild pH, responsible for demineralization, adequate wettability, and diffusion of adhesive monomers for micromechanical retention, showing similar performance to those of G3 and G2 with respect to adhesive strength [1, 18].

G2 had the lowest average bond strength (11.2180 MPa), being statistically different from that of G3 and similar to the bond strength of G1. Groups 3 and 2 had in common the presence of the monomer HEMA. The functional group of this monomer exhibits hydrophilic properties, aiming to improve dentin wetting and demineralization, being considered an adhesion promoter since it contributes to the increase in the bond strength of adhesives to dentin [1, 3, 19–22]. On the other hand, the high hydrophilicity of this monomer promotes water absorption and, therefore, hydrolysis in the adhesive interface, probably affecting the bond strength [2, 23, 24]. Therefore, high concentrations of HEMA in an adhesive can have deteriorating effects on the mechanical properties of the polymer, resulting from gradual hydrolytic degradation [21, 25]. Due to the disadvantages of HEMA, less hydrophilic adhesives without HEMA have been incorporated in the market, which may have reduced water absorption and promoted greater stability of mechanical properties and the interfacial bond [26, 27]. Although the formulation of G3 contained this monomer, the 10-MDP present in its composition probably favored the mechanical properties of this group, showing a statistical difference in comparison to G2, whose formulation contained only the monomer HEMA.

When compared to G1 (HEMA-free), G2 (contained HEMA) was statistically similar, agreeing with the study conducted by Collares [28] who concluded that the HEMA content in the adhesive resin did not influence the bond strength to dentine after 24 hours and after six months. On the other hand, the present study disagrees with the results of the study conducted by Mohammed [29], which indicated that the presence of HEMA improved bond strength to dentin.

G3 had the highest average bond strength (15.6080 MPa), which can be explained by being an adhesive with soft pH, but mainly by the presence of the functional monomer 10-MDP in its formulation. The literature has reported that adhesives with soft pH have been preferred as a dentin bonding agent [11, 30], given that they do not fully remove hydroxyapatite, leaving calcium available for additional chemical interaction with functional monomers [31, 32] that have chemical affinity for hydroxyapatite (like 10-MDP), thus forming more stable bonds [11, 30, 33]. Adhesives based on 10-MDP chemically bond to dentin hydroxyapatite crystals through the electrostatic interactions of ionic bonds formed with calcium ions, and this bond results in an insoluble MDP-Ca salt. Furthermore, the phosphate groups of 10-MDP form covalent bonds with the corresponding phosphate groups of the hydroxyapatite crystals to also form insoluble salts [34, 35]. The unique chemical structure of 10-MDP and the resulting intense and stable adhesion to calcium in hydroxyapatite has been shown to contribute to bond durability, thus enhancing the adhesive performance of self-etching systems [14, 36, 37].

This monomer seems to be a safe choice due to its hydrophobic molecular structure favorable to adhesion, in addition to the characteristics of the adhesive interface formed that favors the durability and strength of the bond with the formation of a water-insoluble MDP-Ca salt [38]. The continuous deposition of successive layers of these salts on the outer surface of the hydroxyapatite crystals is a process known as “nano-layering” [8, 39]. This way, there is a double bonding mechanism, and this chemical interaction with the dental substrate results in greater bond strength when compared to only micromechanical adhesion [7, 40, 41]. The findings of the present study are in line with those of a systematic review of *in vitro* studies [42], which indicated that adhesives containing 10-MDP had superior bonding performance in comparison with materials formulated with other acidic ingredients.

One study revealed that HEMA could significantly affect the chemical interaction between 10-MDP and hydroxyapatite. When the monomer HEMA was added, the MDP-Ca salt formation was remarkably decreased, significantly reducing the nano-layer. Therefore, HEMA could interfere, but did not completely inhibit MDP from interacting chemically with hydroxyapatite [43]. The findings of the study conducted by Yoshida (2012) are in line with the results of the present study, as the G3 that contained the interaction of these two monomers had higher mean bond strength, both with respect to the group containing only HEMA and the group whose formulation did not contain either of these two functional monomers.

On the other hand, the study by Zhou et al. [44] showed that despite the excellent diffusion and apparent higher degree of copolymerization, 10-MDP reduced the elastic modulus of the interface, suggesting poor polymerization, that is, polymerization linearity related to the long carboxyl chain of 10-MDP. This reduced mechanical integrity of hybridization may also be associated with the inhibition of nanolayers between 10-MDP and mineralized tissue in the presence of HEMA. This potential disadvantage of HEMA necessitates further qualitative/quantitative characterization of adhesive-dentin hybridization using an experimental-free or low HEMA 10-MDP monomer, which theoretically has a higher chemical binding potential than the current HEMA-rich protocol. A recent study de 2021 [45] showed that the HEMA monomer can be substituted for the diethyl acrylamide comonomer without changing the bonding strength.

In addition to the properties of functional monomers, there are other important factors to be discussed, such as the number of steps. In the present study, bond strength rates were lower for the two single-step adhesives (G2 and G1) and higher for the two-step adhesive (G3). Simplified one-step adhesives, also called “all-in-one,” theoretically combine the three steps in a single bottle (conditioning, primer, and adhesive), mixing hydrophilic and hydrophobic monomers and therefore requiring a relatively high concentration of solvent to keep them in solution [46, 47]. Due to their high hydrophilicity, one-step self-etching adhesives behave like semi-permeable membranes, allowing the passage of fluids [5, 48], which can jeopardize the adhesive bond. On the other hand, a systematic review with the meta-analysis of clinical studies indicated that one adhesive strategy was not better than the other, regardless of the number of steps [49]. This review agreed with another systematic review, which showed that there was no difference in the retention rates of 3-step wash/dry adhesives and 1-step self-etching adhesives [50]. The mode of application is also important in the adhesive process. Agitation and application time influence the bond strength of self-etching adhesives to dentin [51]. In addition, the air pressure in solvent evaporation can influence the bond strength of universal adhesives [52].

The main limitation found in the present study was the fact that there were no commercially available adhesives that contained only the monomer 10-MDP in their formulation (free of HEMA) to assess the clinical performance of this monomer in isolation. It is also known that the mouth is a hostile environment with acidic and mechanical challenges that cannot always be reproduced in *in vitro* studies. There is therefore a need to measure not only “immediate” but also “aged” bond strength to allow predictions of long-term clinical performance [53].

Monomer 10-MDP seems to be promising with respect to bond stability, whereas HEMA may compromise long-term bond strength. However, adhesive systems are made up of a mixture of monomers, solvents, initiators, and nanoparticles to promote the adhesive process, making it difficult to hold a single component responsible for successful adhesion. The search for more stable materials in the long term and for the quality of the adhesive bond has been constant. Thus, due to a large number of adhesive systems available in the market and the different components present in each of them, deciding which adhesive can be used represents a real challenge. Several laboratory studies have assessed the bond strength of different adhesives in an attempt to predict their clinical outcomes, but a positive correlation has not been found [54]. Thus, further clinical studies should be encouraged to better understand the bond strength of these systems.

## 5. Conclusions

The self-etching adhesive containing the monomer 10-MDP was revealed to be promising in increasing the bond strength between the dental substrate and the composite resin, whereas HEMA exhibited lower bond strength to dentin.

## Figures and Tables

**Table 1:** Experimental groups (*n* = 16 per group).

Group	Adhesive	Composition	Classification	Application mode
G1 (control)	Prime and bond U Dentsply Sirona, Konstanz, Germany	Bis-acrylic diamine; water; propanol; dihydrogen phosphate methacrylate; penta; bis-acrylic propylamine; camphorquinone; hexafluorophosphate; dimethylamino benzonitrile; hydroquinone.	1 step	Apply keeping the surface completely wet; if necessary, apply twice. Shake lightly for 20 seconds. Apply a moderate blast of air for at least 5 seconds to keep the layer even. Ten- to twenty-second curing time

G2	OptiBond All-in-One Kerr, New South Wales, Australia	Acetone; 2-hydroxyethyl methacrylate; ethyl alcohol; 2002Dhydroxy-1,3-propanediyl bismethacrylate; silica; amorphous, fumed, cryst-free, methyl alcohol.	1 step	Active application of the first layer of adhesive for 20 seconds. Active application of the second layer of adhesive for 20 seconds. Dry with light blast and then medium blast for at least 5 seconds. Ten-second curing time.

G3	Clearfil SE Kuraray Medical Inc, Tokyo, Japan	Primer: MDPB; MDP; HEMA; hydrophilic dimethacrylate; photoinitiator; water Bond: MDP; HEMA; Bis-GMA; hydrophobic dimethacrylate; photoinitiators; silanates.	2 step	Active application of primer for 20 seconds. Short air blast for 5 seconds. Active application of bond for 15 seconds. Removal of excess with an air blast for 3 seconds. Ten-second curing time.

**Table 2:** Results of the experimental groups (MPa).

	Experimental groups (*n* = 16 per group)		
	G1	G2	G3
Median	14.6325^AB^	11.2180^A^	15.6080^B^
Interquartile deviation	(±5.36)	(±3.91)	(±5.14)

*Note.* Different letters indicate statistical difference (5%).

## Data Availability

The data are given in references.
